# High-resolution patterning of solution-processable materials via externally engineered pinning of capillary bridges

**DOI:** 10.1038/s41467-018-02835-7

**Published:** 2018-01-26

**Authors:** Shunpu Li, Young Tea Chun, Shuo Zhao, Hyungju Ahn, Docheon Ahn, Jung Inn Sohn, Yongbing Xu, Pawan Shrestha, Mike Pivnenko, Daping Chu

**Affiliations:** 1 Centre for Photonic Devices and Sensors, University of Cambridge, 9 JJ Thomson Avenue, Cambridge, CB3 0FA UK; 2 Department of Electronic Engineering, University of York, Heslington, York, YO10 5DD UK; 3 Beamline Department, Pohang Accelerator Laboratory, POSTECH, Gyeongbuk, Pohang, 37673 Republic of Korea; 4 Department of Engineering Science, University of Oxford, Parks Road, Oxford, OX1 3PJ UK; 5 York-Nanjing Joint Centre for Spintronics and Nano Engineering (YNJC), Nanjing University, Nanjing, 210093 China

## Abstract

Electronics based on solution-processable materials are promising for applications in many fields which stimulated enormous research interest in liquid-drying and pattern formation. However, assembling of structure with submicrometre/nanometre resolution through liquid process is very challenging. We show a simple method to rapidly generate polymer structures with deep-submicrometre-sized features over large areas. In this method, a solution film is dried on a substrate under a suspended flexible template with groove/ridge surface topography. Upon solvent evaporation, the solution splits in the grooves and forms capillary bridges between the template and substrate, which are firmly pinned by the edges of the template grooves. This groove pinning stabilizes the contact lines, thereby allowing the formation of fine patterned structures with high aspect ratios which were used to fabricate various functional materials and electronic devices. We also produced secondary self-assembled nano-stripe patterns with resolutions of about 50 nm on the primary lines.

## Abstract (web summary)

Solution-processed electronics is desirable for large-scale electronics, but it is challenging to fabricate nanometre patterns via liquid processes. Li et al. address this problem using soft molds with groove and ridge structures, which control thin-film morphology upon dying via the pinning effect.

## Introduction

Liquid flow/drying-induced edge deposition from solution/suspension is frequently observed in daily life. For instance, natural levees formed by the deposition of sediments along flooded river banks have been utilized for settlement and agriculture since ancient times^1,2^. The drying of a solution deposited on a solid surface often results in a dense, ring-like solute deposit along the perimeter, which is known as the coffee-stain effect^3,4^. Recent developments in thin-film coating, printing, and solution-processable electronics have revived interest in micrometre/nanometre-scale drying processes^5–7^, and considerable efforts have been made to generate high-resolution patterned structures by controlling solvent evaporation^8,9^. Several research groups have generated structures with small features via the controlled drying of solution through digital deposition, including drop-wise deposition using inkjet printing and line-wise deposition by pulling a sharp blade^10–14^. It is desirable to produce such fine structures over large areas with improved resolution using a rapid approach (e.g., a stamp-like technique). Attempts have been made to control liquid evaporation in a confined geometry by holding the solution in between a substrate and a cover object. Under a parallel flat coverage without surface structure, the drying does not produce any regular patterns as demonstrated in previous works^15,16^. Thus, attempts have been made to control liquid evaporation in a confined geometry by retaining the solution between a substrate and a cover object with specific shape. For example, gradient concentric ring patterns can be generated by drying a solution confined between a flat substrate and spherical surface^8,17^. However, several issues related to the use of these patterns, including feature size, limited pattern geometry, pattern instability, and low aspect ratio, must be addressed before they are applied in practical applications. Fine structure fabrications with stamp-guided drying through progressively shrinking capillary bridges were reported^18,19^. In this study, we demonstrate an approach with different drying mechanism to fabricate high-resolution structures via solvent evaporation in confined geometries. A surface-structured flexible template is used to pattern the liquid into capillary bridges and further guide the liquid-drying process with liquid bridge pinning. This work was primarily motivated by the groove pinning of the contact line, which was theoretically proposed several decades ago for drying liquids on grooved substrates;^20,21^ however, in this study, the grooves are patterned on a flexible cover object rather than a substrate. This cover object effectively guides pattern formation on the substrate and can be reused. The microgrooves on the applied template firmly pin the patterned liquid, which is held by capillary force between the substrate and template. This stabilizes the contact line between the liquid and the substrate to form pinned liquid walls, favouring nearby solute deposition with high aspect ratio and high resolution. We show the applicability of this technique with produced high-resolution patterns of various materials and fabricated polymeric electronic devices.

## Results

### Principle of patterning and demonstration

**Fig. 1:**
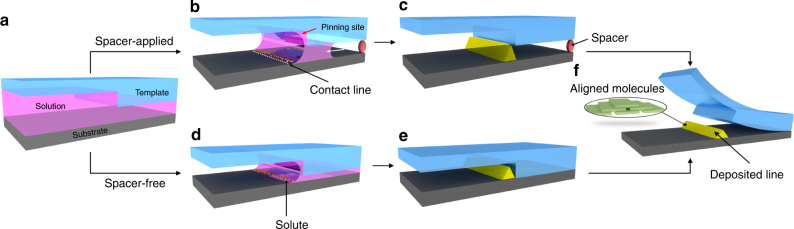
Schematic illustration of the pattern-formation process. The solution is patterned and pinned by the groove corners during drying. Spacer-applied (**a**→**b**→**c**) and spacer-free (**a**→**d**→**e**) configurations can be used to fabricate fine structure through solute transfer to the pinned contact line via capillary flow. For convenience, only half of one ridge and groove is drawn. The molecular alignment and backbone packing with face-on configuration in the generated lines are presented in **f**

The pattern-formation principle is schematically illustrated in Fig. 1. A layer of solution is introduced between a flexible microstructured template and a substrate and dried at an appropriate temperature (Fig. 1a). As solvent evaporation progresses, the solution splits in the groove, and the formed liquid surface migrates towards the sidewall of the groove; thus, the solution is patterned into capillary bridges suspended between the substrate and template (Fig. 1b). The solution splitting in the grooves and capillary bridge formation process is illustrated in Supplementary Figure 1. Because the template is well wetted with organic solvent, a tiny amount of solution is trapped in the corner of the groove^22,23^ and merges into the capillary bridges. Thus, the contact line between the liquid bridge and the substrate is pinned by the groove. The liquid that evaporates from the edge is replenished by liquid from the interior; the resulting outward flow carries solute to the edge, while solute in the bulk solution underneath the ridge does not precipitate because evaporation from the top surface is inhibited. To manipulate the capillary bridges, spacers with submicrometre heights can be directly fabricated on the template surface, controlling the gap between the template and substrate (red circle in Fig. 1b, c). However, spacers are not always necessary because a liquid film is often trapped between the template ridges and substrate when a template is attached to a wet substrate (about 700 nm for isopropanol (IPA)-based and 1,2-dichlorobenzene (DCB)-based solutions was estimated). In the absence of spacers, the template shifts downward as the solvent evaporates, eventually coming into contact with the substrate when drying is complete (Fig. 1d, e). If spacers are applied to suspend the template, the capillary bridge underneath the template ridge will split and is dragged gradually towards the groove as solvent evaporates further (white arrow in Fig. 1b). In this case, the solute is deposited only at the groove-pinned contact lines; no deposition occurs next to the capillary walls under the ridges because they are not pinned. We fabricated patterns both with and without spacers (denoted as spacer-applied and spacer-free configurations, respectively). In fabrication experiments, a controlled volume of solution (about 3 μl for a template with a 1 cm^2^ pattern area) was drop-casted onto the surface of a structured polydimethylsiloxane (PDMS) template. Then, a substrate (e.g., a silicon wafer) was gently brought into contact with the solution-wetted template and dried at room temperature for 90 min under a small applied pressure (about 5 MPa). Finally, the template was removed, leaving the patterned polymer on the substrate (Fig. 1f). The depths of grooves on the masters for duplicating PDMS templates were 1.5 μm (photoresist) and 800 nm (poly(methyl methacrylate) (PMMA)) for the two types of masters made with photolithography and electron-beam (e-beam) lithography, respectively.

**Fig. 2:**
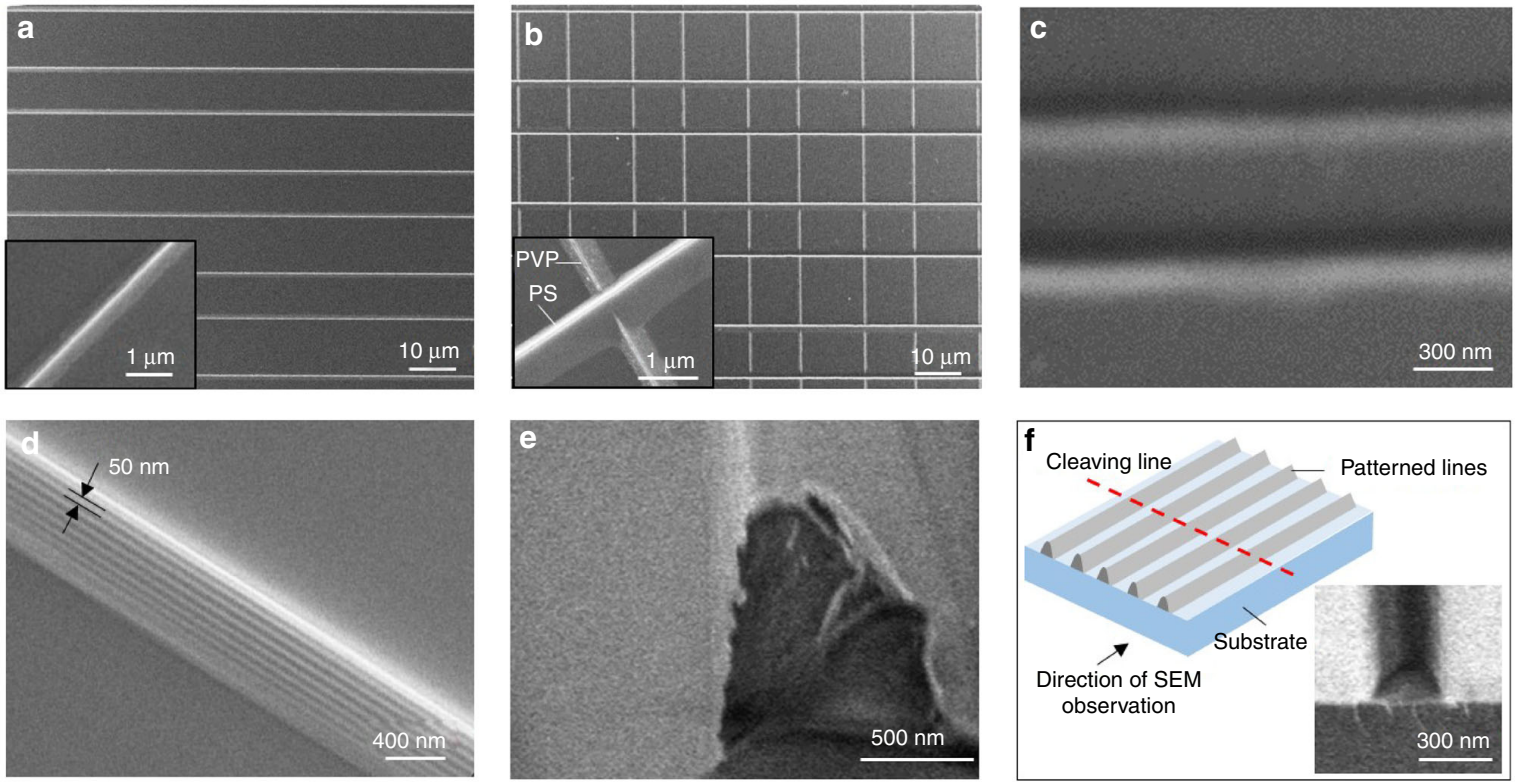
Scanning electron microscopy images of patterned structures. **a** Line pattern of 2008P, a polymeric semiconductor. **b** Grid structure composed of sequentially patterned PVP and polystyrene lines. **c** Fabricated PS lines with about 300 nm resolution and 150 nm feature. **d** Self-assembled secondary pattern (resolution around 50 nm) on a primary PS line. **e**, **f** SEM images of cross sections of patterned PS lines with different feature sizes and schematic drawing to illustrate of how the sample was cleaved and imaged

Figure 2a shows lines of poly(9,9-dioctylfluorene-*alt*-bithiophene) (2008P), a *p*-type semiconductor polymer, patterned using a spacer-free configuration from DCB solution (2.5 mg ml^−1^). To confirm the role of the liquid bridges, we performed patterning using a spacer-applied configuration in which a first layer of patterned lines produced using a spacer-free configuration served as spacers for patterning a second layer. First, we created patterned lines of poly-4-vinylphenol (PVP) from its IPA solution (2.5 mg ml^−1^). The PVP pattern was then used as a spacer to further pattern polystyrene (PS) from DCB solution (2.5 mg ml^−1^). Figure 2b shows an image of the produced grid consisting of perpendicularly oriented PVP and PS lines. The lines are jointed at cross-points so that the morphology of lines created by the second patterning was not influenced by the existing lines from the first patterning. Both the PVP and PS lines have similar morphologies originating from the similar mechanism of line formation: capillary flow induces polymer deposition next to the walls of the hanging liquid bridges to form triangular prism-shaped polymer lines as a result of geometric restriction. Such sequentially deposited grid structures are interesting for many applications, like optical and hierarchical materials and so on^24^. The experiments demonstrated that the proposed process is robust and capable of producing uniform patterns over large areas (Supplementary Figure 2). The obtained pattern area was 10 mm × 10 mm and was limited by the available template size. The feature size of the fabricated structure can be varied from about 150 nm to several micrometres by tuning the solution concentration (Fig. 2c and Supplementary Figures 3–5). To investigate the influence of concentration, solutions with various polymer concentrations ranging from 0.2 to 70 mg ml^−1^ were tested. When the polymer concentration exceeded the critical concentration (about 30 mg ml^−1^ for PS from DCB and about 50 mg ml^−1^ for PVP from IPA), clear patterns were not obtained due to the precipitation of residual polymer under the ridges of the template (Supplementary Figure 3c and Supplementary Figure 4c), which occurred because the polymer concentration crossed over the binodal curve in the corresponding phase diagram^25,26^. The effect of line separation on pattern quality was also investigated (Supplementary Figures 6–8). The minimum line period (sum of width and separation) we obtained was 600 nm (Fig. 2c), which is superior to those achieved by other patterning methods based on solution drying (e.g., the coffee-stain effect of inkjet printing; Fig. 2c)^11^. For a solution with a concentration of 2.5 mg ml^−1^, the separation between patterned lines can be larger than 100 μm with no observable residual polymer contamination between the lines. The cleanness of the interline spaces is supported by atomic force microscope (AFM) and energy dispersive x-ray (EDX) analysis (Supplementary Note 1 and Supplementary Figure 9).

Unexpectedly, we also obtained a secondary pattern with high resolution (about 50 nm) decorated on the walls of the primary patterned lines described above. Figure 2d shows one such nano-sized secondary stripe pattern on a primary PS line. Such secondary stripe patterns only appeared on the sidewalls facing the groove walls of the template when patterning was conducted using the spacer-free configuration. The formation of such self-assembled, regular patterns of single-phase materials using solution processes is rare, and the pattern resolution observed here is the highest reported to the best of our knowledge. Liesegang rings, which were first observed a century ago, typically have resolutions on the order of millimetres^27^, while evaporation-induced concentric rings formed from solution in a sphere-on-flat-surface geometry have micrometre-sized features^9^. We attribute the observed nanopatterns to repeated pinning and de-pinning events during the late stage of drying. When the template ridges reach the substrate a tiny amount of solution becomes trapped in the wedge-shaped space between the groove sidewall and the newly formed primary polymer line (Supplementary Note 2 and Supplementary Figure 10). The detailed formation process of these secondary patterns requires further investigation.

Finally, the cross-section SEM images clearly show a Gaussian-like profile of the deposited lines with high aspect ratio (Fig. 2e, f). The aspect ratio varies with line feature size, for instance, a 1 μm featured line has aspect ratio around one (1 μm footprint, 1 μm height), while for a 300 nm featured line the aspect ratio is around 0.6 (300 nm footprint, 180 nm height).

### Evaluation of polymer wire patterns

**Fig. 3:**
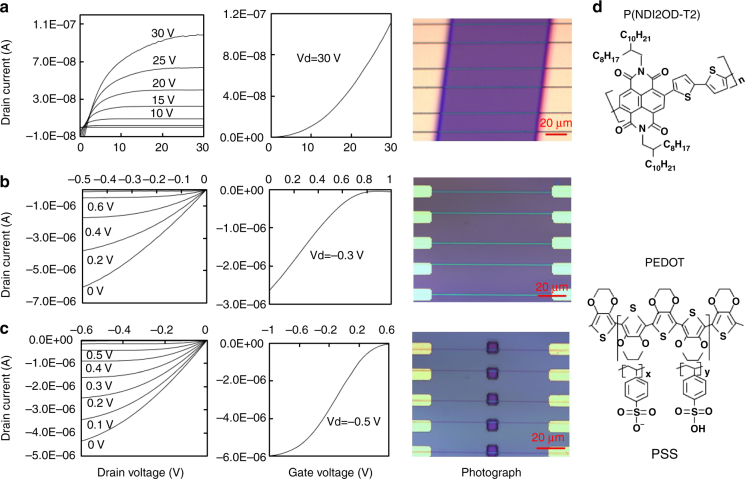
Performances and images of devices, and molecule structures. **a** Top-gated FET with n-type P(NDI2OD-T2) wires. **b** Array of electrochemical transistors with PEDOT:PSS wires. **c** Electrochemical transistors with PEDOT:PSS wires covered by patterned photoresist for local sensing. **d** Molecule structures of P(NDI2OD-T2) and PEDOT:PSS polymers

To demonstrate the potential of the developed process, we fabricated different types of transistors with polymer wires as active materials which is interesting for various applications, like brain mapping and synaptic devices with low energy consumption^28–30^. Figure 3a shows the performance of a top-gated field-effect transistor (FET) comprising an array of poly{[*N*,*N*0-bis(2-octyldodecyl)-naphthalene-1,4,5,8-bis(dicarboximide)-2,6-diyl]-*alt*-5,5′-(2,2′-bithiophene)} (P(NDI2OD-T2)) wires (about 900 nm in width) formed from DCB solution (3 mg ml^−1^) on a SiO_2_ (300 nm)/Si substrate with patterned Au source-drain electrodes. Spin-coated PMMA and thermally evaporated Al were used as the dielectrics and gate electrode, respectively. The charge-carrier mobility of the device was 0.34 cm^2^ V^–1^ s^–1^, which is several times higher than that of the spun-cast device (0.054 cm^2^ V^–1^ s^–1^) fabricated with identical process as that of the wire devices, except the deposition step of the semiconductor layers (Supplementary Figure 11). The charge mobility (*μ*) was calculated using the expression^31^ with a small modification, that is, deduction of the interline space area by replacing channel width *W* with *aW λ*^−1^, to suit the nature of our devices:

1 $${\mathrm{\mu }} = \frac{{2\lambda {\it{Ld}}I_{\mathrm{D}}}}{{W\varepsilon _{\mathrm{r}}\varepsilon _0a(V_{\mathrm{G}} - V_{\mathrm{T}})^2}},$$

where *a* and *λ* are the width and period of the patterned semiconductor wires, respectively, *W* and *L* are the channel width and length of the electrode, respectively; *ε*_r_ and *d* are the relative permittivity and thickness of the dielectrics, respectively; *ε*_0_ is the vacuum permittivity; and *I*_D_, *V*_G_ and *V*_T_ are the drain current, gate voltage and threshold voltage, respectively. The high aspect ratio of the generated lines facilitates the transfer of the patterns onto other materials using standard semiconductor techniques such as plasma etching.

To demonstrate the transfer of the formed patterns, we fabricated arrays of electrochemical transistors with individually electrically addressed wires of active materials. Poly(3,4-ethylenedioxythiophene)-poly(styrenesulfonate) (PEDOT:PSS) was chosen as the active material because it is robust to photolithography processes, and PEDOT:PSS-based devices have promising applications in electrophysiological recording^29^. An array of PEDOT:PSS wires was created by transferring the pattern from an array of PS lines generated using our method onto a spin-coated PEDOT:PSS film via plasma etching. Au source-drain electrodes were fabricated by optical lithography and subsequent lift-off (Fig. 3b), and silver conducting wire and 0.1 M NaCl aqueous solution were used as the gate electrode and electrolyte, respectively, during device characterization. A millimetre-sized PDMS frame was physically attached on the sample surface to confine the electrolyte. The transistors were evaluated under small applied gate and drain voltages (<0.5 V). The drain current decreased as the gate voltage increased (i.e., working with depletion model because of the partial balance of the negatively charged PSS^–^ by Na^+^, thus, PEDOT was de-doped). The potential of this wire-based PEDOT:PSS electrochemical transistor was further demonstrated through a local sensing experiment. We masked the device with a photoresist film, leaving only a small hole to expose a tiny segment of each PEDOT:PSS wire to the electrolyte (Fig. 3c). The single-wire device performed well, even when the doping and de-doping process occurred only locally, which is crucial for high-resolution sensing (e.g., bio-recording), where the ion concentration varies within the subcellular domain. For clarity, the chemical structures of P(NDI2OD-T2) and PEDOT:PSS are shown in Fig. 3d.

**Fig. 4:**
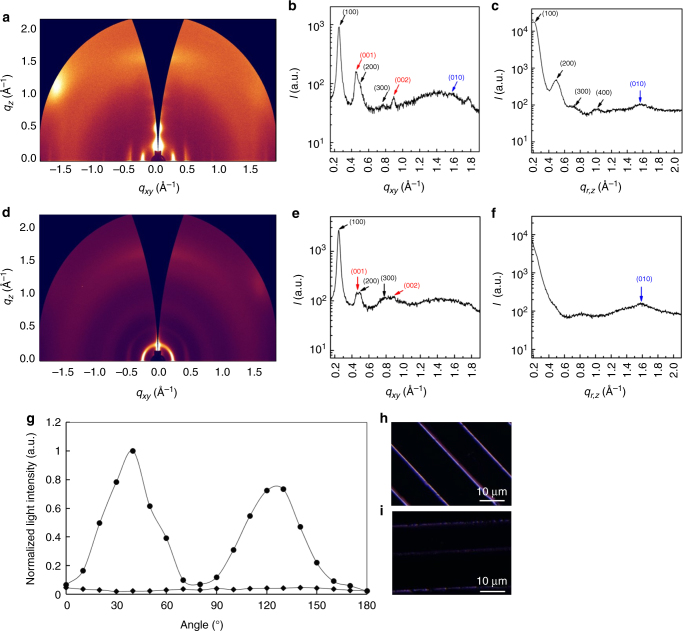
Evaluation of the patterned wires. GI-WAXD results for a spun-cast P(NDI2OD-T2) film (**a**–**c**) and patterned P(NDI2OD-T2) wires (**d**–**f**). **b**, **c** The in-plane and out-of-plane data of the spun-cast sample, respectively, while **e** and **f** are the in-plane and out-of-plane data of patterned wires taken in the wire direction. Both devices were annealed at 140 °C for 30 min. Angle dependence of the brightness of patterned P(NDI2OD-T2) lines (**g**) measured under polarized microscope for samples created using our patterning method (solid circles) and by plasma etching a spin-coated P(NDI2OD-T2) film with PVP lines as a mask (solid diamonds). **h**, **i** Birefringence images of wires with different orientations around 45° and 180°, respectively

The high charge mobility of the produced P(NDI2OD-T2) wires originates from the favourable backbone packing and polymer chain alignment induced by the hydrodynamic process during wire formation, as confirmed by grazing incident wide-angle x-ray diffraction (GI-WAXD) and polarized microscopy. Molecular orientation plays a significant role in charge transfer in organic semiconductors^32,33^. Figure 4a–f show the two-dimensional (2D) GI-WAXD patterns and line-cut intensity profiles along the in-plane and out-of-plane directions for the spun-cast film and patterned wires. In the GI-WAXD pattern of the spun-cast film, (n00) diffraction peaks are observed for both the in-plane and out-of-plane directions, indicating a mixed packing structure with both edge-on and face-on orientations in the spun-cast film. In the intensity profiles, (100) and (010) diffraction peaks attributed to lamellar packing and π–π staking are observed at *q*_*xy*_ or *q*_*z*_ = 0.249 Å^–1^ (*d*_100_ = 25.2 Å) and 1.6 Å^–1^ (*d*_π–π_ = 3.92 Å). A (001) diffraction peak attributable to the repeating of chain backbone^34^ is also observed at *q*_*xy*_ = 0.45 Å^–1^ (*d*_001_ = 14.0 Å). In the case of the patterned wire, (n00) and (00n) diffraction peaks are observed along the in-plane direction in the 2D GI-WAXD pattern and in-plane intensity profiles. In contrast, (010) peaks are only observed in the out-of-plane data, and the (n00) peaks in the out-of-plane direction are significantly weaker and broader compared to those of the spun-cast film. This indicates that the molecules in the pattered wires are predominantly packed with face-on orientations. Based on the azimuthal scans of the (200) diffraction peaks, we estimated the proportions of molecules with face-on orientations to be approximately 48 and 70% for the spun-cast and patterned samples, respectively. Rivnay et al. ^34^ reported enhanced charge mobility in organic FETs with primarily face-on molecular orientations, in agreement with our experimental results.

Figure 4g shows the angle dependence of the brightness of patterned P(NDI2OD-T2) lines measured under polarized microscope for samples fabricated using our patterning method and samples produced by plasma etching a spin-coated P(NDI2OD-T2) film with PVP lines as a mask. Figure 4h, i are birefringence images of wires with different orientations around 45° and 180°, respectively. The P(NDI2OD-T2) lines generated by our patterning method clearly show strong birefringence, indicating that the polymer chains are aligned in the direction of the wires.

### In situ investigation of the formation of a polymer wires

**Fig. 5:**
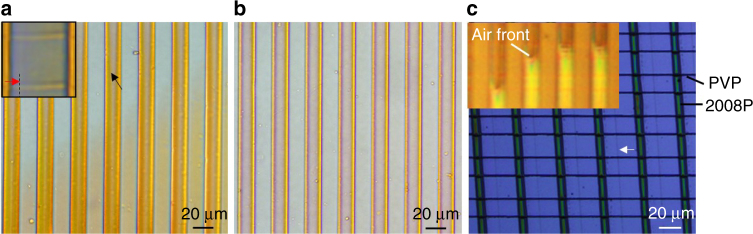
In situ analysis of drying process. The optical images of the drying of a solution of 2008P semiconducting polymer in DCB under a PDMS template at 2 min (**a**) and 10 min (**b**) from sample loading. The black arrow indicates liquid splitting in the grooves of the template, and the red arrow in the inset of **a** indicates a liquid boundary (partially marked by the dotted line) under a template ridge. The two horizontal lines in the inset of **a** indicate the PVP spacer used when 2008P lines were created with the spacer-applied configuration. **c** Optical image showing the de-pinning of the contact line when groove pinning is lost. The white arrow indicates the original contact line between the silicon substrate and 2008P solution bridges. The horizontal lines are the PVP spacers for the patterning of 2008P. The inset of **c** shows air fronts moving in the grooves during drying in the spacer-applied configuration

To investigate the drying process, pattern formation was tracked using in situ optical images. Because of the colour of the 2008P polymer semiconductor, the groove-pinning mechanism was evident when the drying was investigated under a microscope with transmitted light. Figure 5a shows an image of a sample with the spacer-free configuration on a glass substrate taken at an early stage of drying (2 min after sample loading); the image clearly shows the liquid splitting and migrating towards the sidewalls of the grooves (indicated by a black arrow). A similar event was observed in the spacer-applied configuration when pre-patterned PVP lines were used as the spacers for the patterning of 2008P. Splitting of the liquid film underneath the ridges of the template occurred in the samples with the spacer-applied configuration (red arrow in the inset of Fig. 5a).

The organic solvents used in this study (IPA and DCB) wet the PDMS well and tended to dwell in the corners of the grooves because corners provide a larger liquid/solid interface than flat surfaces and are thus favourable for energy minimization^22,23^. According to the Concus–Finn relation, a liquid droplet will spread in a corner with half-angle *β* formed by two solid walls if the contact angle on both walls *ϑ* satisfies^35^

2 $$\vartheta < \frac{\pi }{2} - \beta .$$

Based on Equation (2), *ϑ* *<* 45° is required for our experiment; the measured contact angles (*ϑ* *=* 28.7° for IPA/PDMS and *ϑ* *=* 38.9° for DCB/PDMS) satisfy this requirement. Figure 5b shows an in situ image taken from the same sample shown in Fig. 5a at a later stage of drying (10 min after sample loading), revealing fine 2008P lines deposited next to the sidewalls of the grooves. Groove pinning is the primary mechanism for forming such fine, high-aspect-ratio patterns. Although the substrate itself also contributes to contact line pinning, if groove pinning does not occur at one side of a template ridge, the pinning strength of the substrate alone is not sufficiently strong to pin the liquid, and the solution is dragged under the template ridge by capillary force. Figure 5c shows such a failed patterning of 2008P using the spacer-applied configuration with PVP lines as spacers on a Si substrate. Fine polymer lines clearly formed before the onset of de-pinning (indicated by the white arrow in Fig. 5c). Failed patterns such as this were occasionally observed in our experiments and were likely caused by the structural deformation of the template as a result of shearing force, which can be avoided by optimizing a number of factors, like mechanical properties of the template materials, designs of the groove profile and the experimental system and so on. From Fig. 2b and Fig. 5c we see that a sharp groove profile is essential to pin the contact lines. This is in contrast to previously reported work where stamps with structures of spherical domes were applied to insure the shrinking of capillary bridges with the solvent evaporation (i.e., the contact line is unpinned)^19^. We have also conducted experiments with PDMS template without grooves where polymer solutions were dried between blank PDMS films and Si substrates with and without micronmetre-sized spacers. No regular line patterns were created on the Si surface (Supplementary Note 3 and Supplementary Figure 12). This further proves the importance of the grooves on PDMS to pattern and pin the solution.

Comparing with liquid drops on a flat solid surface, the micro-liquid bridges formed using our method are more favourable for solute transfer to the pinned contact line because evaporation from the top surface is restricted, preventing precipitation in the far field of contact line. Thus, the resulting outward flow can carry all of the dissolved material to the pinning lines. For the template used in this study (12 mm × 12 mm with a 10 mm × 10 mm patterned area), we could not clearly observe how air is introduced from the external environment during drying using optical microscopy. Both external air and air trapped in the template materials can contribute to solvent extraction^36^. Air trapping from the external environment could be clearly observed with reflected light under a microscope when the PDMS template was cut along the direction perpendicular to the grooves in the structured area (inset of Fig. 5c). A video of liquid splitting in microgrooves during drying can be found in the Supplementary Information movie 1 and 2. We chose DCB and IPA as solvents in our experiments because a high-boiling-point solvent allows for sufficient implementation time. Drying the samples at elevated temperature or using a solvent with a low boiling point allows the patterning to be completed in minutes, revealing the potential to scale-up the patterning process. The rapid pattern formation is attributed to efficient solvent extraction during drying. To obtain more information about solvent extraction, we dried PS from DCB solution on a silicon substrate with the spacer-free configuration at various temperatures and measured the advancing speed of the air fronts in situ. We found that for a given temperature, the advancing speed of the front of inletting air is constant regardless of its location within the sample; we expected the advancing speed of the air front to decrease when the air front was propelled into the deep side of the sample if the solvent extraction was controlled by vapour diffusion. We found the speed *V* and drying temperature *T* (in Kelvin scale, *K*) to be exponentially related (Supplementary Note 4 for details):

3 $$V = V_0\exp \left( { - Q/kT} \right),$$

where *Q* and *k* are the activation energy and Boltzmann constant, respectively. By fitting our experimental *V–T* curve (Supplementary Figure 13) using Equation (3), we determined the activation energy *Q* *=* 5.96 × 10^−20^ J and enthalpy of vaporization of the solvent *E* *=* *N*_a_*Q* *=* 36,000 J mol^−1^, where *N*_a_ is Avogadro’s constant. This value of *E* is in good agreement with the chemical data sheet value of 39,400 J mol^−1^. Thus, the solvent drying in this experiment was controlled by the energy required for a solvent molecule to escape from the liquid surface rather than by diffusion. In other words, the solvent vapour can be quickly extracted once it leaves the liquid surface. The value of *V*_0_ is about 250 μm s^−1^ which represents the up-limit of the moving speed of the air front in the grooves (i.e., when the temperature is very high).

The method presented in this paper can be used to fabricate structures and materials that cannot be easily generated by conventional methods. For example, we fabricated fine polymer structures on curved surfaces (e.g., inner and external surfaces of glass tubes with diameters of 6 mm; Supplementary Note 5 and Supplementary Figure 14) and patterned DNA molecules while avoiding heating and irradiation (Supplementary Note 6 and Supplementary Figure 15) similar to that described in Byun et al.^19^. We also patterned ZnO nanoparticles from a colloidal suspension using the spacer-applied configuration (Supplementary Note 6 and Supplementary Figure 16). No obvious impact on the feature of the patterned lines has been observed from PDMS swelling. The obtained Gaussian line shape is symmetric, although asymmetric line shape can be observed occasionally which might be caused by PDMS swelling. However, the impact of the PDMS swelling on interline distance is observable which is induced by changing the dimensions and shape of the protrusions of the patterned PDMS^37,38^. Template material other than PDMS, like NEA123L, a UV-curable adhesive, was also successfully applied to pattern polymer materials (Supplementary Note 7 and Supplementary Figure 17). Supplementary Table 1 compares our method with conventional patterning techniques. We can see that the fabrication process described here has the potential to fabricate structures with resolution to match that fabricated with many well-developed techniques while it is applicable for certain situations where conventional lithography technologies are not suitable, for instance, avoiding material degradation with UV irradiation and chemical attack during structure development, multistep patterning without material overlap at cross-points.

## Discussion

We have demonstrated a process to generate fine structures with deep submicrometre features through controlled liquid drying. A structured flexible template is used to pattern a liquid film via free energy minimization based on the fact that the groove corners of the template are energetically favourable locations for liquid dwelling. During solvent evaporation, the solution splits in the grooves of the template and migrates towards the groove sidewalls, where it is patterned into capillary bridges that are pinned by the grooves during further drying. This groove pinning stabilizes the contact line between the capillary liquid bridge and the substrate where the solute is deposited. The liquid bridges pinned by the groove sidewalls allow for the formation of fine, self-assembled patterns with high aspect ratios. These patterns are favourable for their subsequent transfer into other functional materials. Comparison to droplets of solution on a flat solid surface, the micro-liquid bridges formed during our process are advantageous for transferring the solute to the pinned contact lines and for avoiding residual formation in the far field of the lines because evaporation from the top surface is restricted. In the newness method, deposition along the contact line of a hanging liquid bridge on the substrate allows the formation of lines with both high-resolution and high aspect ratio.

The newness patterning process is favourable for chain alignment and backbone packing in polymers, as confirmed by GI-WAXD and polarized microscopy. We successfully fabricated transistors with the patterned polymer conductor/semiconductor wires, demonstrating the potential applications of the generated structures. Furthermore, secondary self-assembled nano-stripe patterns with resolutions of about 50 nm are observed on the sidewalls of the formed primary lines. These nano-stripes are many orders of magnitude smaller than previously reported self-assembled patterns formed via liquid processes (e.g., millimetre-sized Liesegang rings and micrometre-sized concentric rings formed from solutions confined in sphere-on-flat geometries).

## Methods

### Template preparation, line forming and in situ observation

The structured PDMS template was duplicated by pouring commercial silicone elastomer (Sylgard^®^184, Dow Corning), supplied as a two-part liquid component kit, with a 10:1 mix ratio onto an optical or e-beam lithography-defined photoresist/PMMA masters and annealing at 70 °C for 1 h. Material patterning was performed on a homemade stainless-steel clamping tool, which provided the proper sample/template alignment, under the application of controlled pressure. For in situ microscopic observation, both transmissive and reflective modes were used. Samples were clamped in transparent plastic boxes with open holes to allow solvent evaporation. Transmissive mode was used to investigate the formation of 2008P polymer (American Dye Source, Inc.) patterns on glass substrates, whereas reflective mode was used to investigate the drying dynamics on Si substrates.

### Device fabrication

To generate PEDOT:PSS wires, a PEDOT:PSS (Clevios PH-1000) water-based suspension from Heraeus was modified by adding 20% ethylene glycol (Sigma-Aldrich) to improve the conductivity and 1% Triton X-100 (Sigma-Aldrich) to reduce surface tension and obtain a highly uniform film. A 90-nm-thick PEDOT:PSS film was formed by spin-coating PEDOT:PSS onto an SiO_2_ (300 nm)/Si substrate and annealing at 140 °C for 1 h. PVP lines were created from its IPA solution using our developed method. After plasma etching with a gas mixture of CF_4_+O_2_(1:1), the sample was rinsed with IPA to remove the PVP lines. Au electrodes for addressing individual PEDOT:PSS lines were produced by optical lithography, followed by the thermal evaporation of Au(80 nm)/Ti(10 nm) and lift-off in acetone. The same process was used to pattern a spin-coated P(NDI2OD-T2) (Polyera Corporation) film for polarized microscopy analysis. A 50-nm layer of P(NDI2OD-T2) was spin-coated from its toluene solution and baked at 100 °C for 30 min. PVP lines were then generated by our method, and the sample was etched with CF_4_+O_2_(1:1) plasma. Finally, the PVP lines were removed in IPA.

For FET fabrication, P(NDI2OD-T2) lines were generated from DCB solution using our method on an SiO_2_ (300 nm)/Si substrate with patterned Au/Cr electrodes. The sample was then annealed at 140 °C for 4 h in N_2_. PMMA dielectrics (thickness of 900 nm) was spin-coated and annealed in N_2_ at 80 °C for 30 min, and an Al top gate was then deposited through a shadow mask by thermal evaporation. The channel lengths and widths of the Au electrodes were 95 and 1750 μm, respectively. Similar process was applied for TFT fabrication with spun-cast P(NDI2OD-T2) film.

### Analysis of molecule conformation and sample quality

GI-WAXD measurements were conducted at the PLS-II 9A U-SAXS beamline of PAL in Korea. The X-rays from the in-vacuum undulator were monochromated (wavelength = 1.11 Å) using a double-crystal monochromator and focused both horizontally and vertically (FWHM = 300 μm (H) × 30 μm (V) at the sample position) using K–B type mirrors. The GI-WAXD system was equipped with a seven-axis motorized sample stage for the fine alignment of thin film. The sample-to-detector distance was 224 mm, and diffraction patterns were recorded with a 2D charge-coupled device detector (Rayonix SX165).

The AFM and EDX analysis were performed on Nanoscope III and LEO GEMINI 1530VP FEG-SEM system, respectively.

### Data availability

The data that support the findings of this study are available from the corresponding author upon reasonable request.

## Electronic supplementary material

Supplementary Information

Description of Additional Supplementary Files

Supplementary Movie 1

Supplementary Movie 2
