# Supplementary material for: High-resolution patterning of solution-processable materials via externally engineered pinning of capillary bridges
Source: Nat Commun. 2018 Jan 26;9:393. doi: 10.1038/s41467-018-02835-7 (PMC5786051; doi:10.1038/s41467-018-02835-7)
Supplement: Supplementary file 1 — Supplementary Information [file 41467_2018_2835_MOESM1_ESM.pdf]

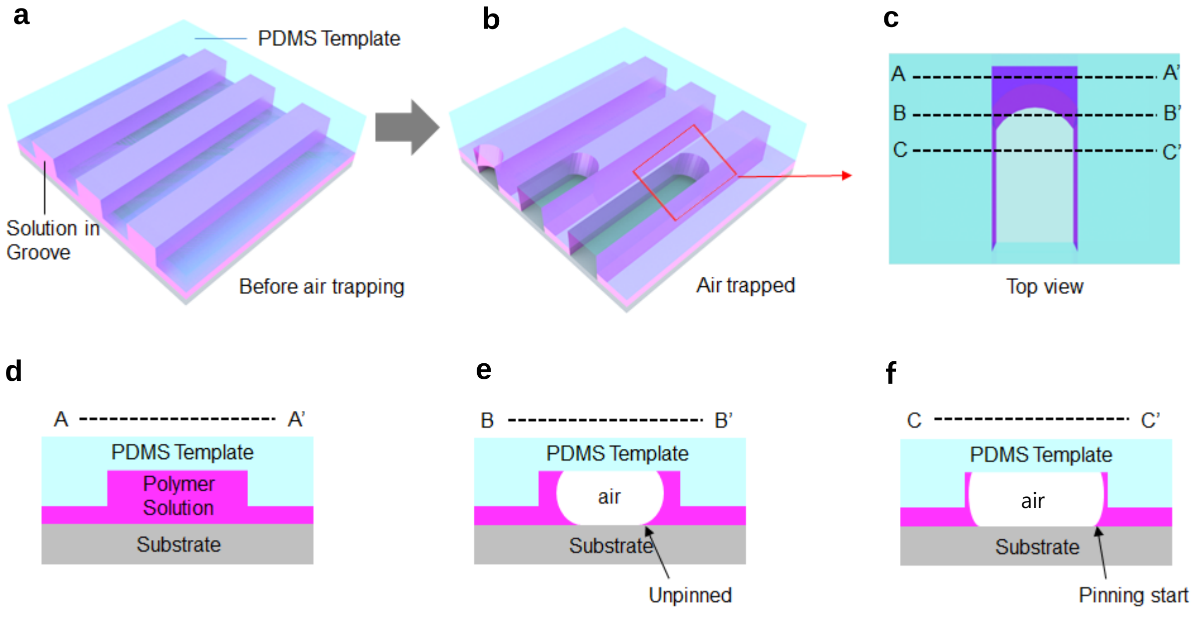

Supplementary Figure 1. Schematic illustration of liquid splitting in grooves. (a) Initial stage when solution is loaded. (b, c) Air is trapped into the grooves. (d-f) Front views of different cross sections as indicated in (c).

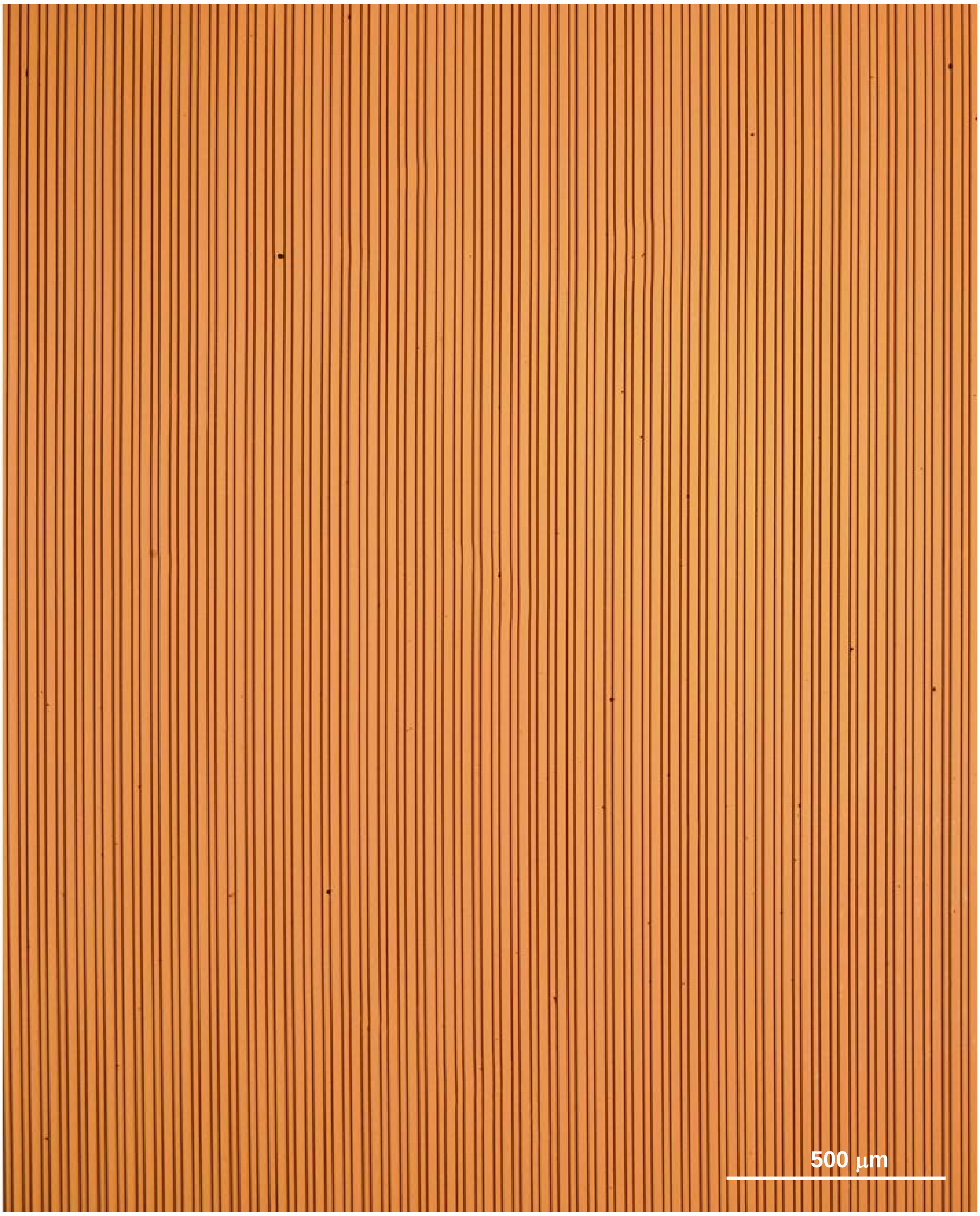

Supplementary Figure 2. Optical image of a polystyrene pattern. The pattern was generated from its 1,2-dichlorobenzene solution (5mg ml^{-1}), and the image covers 2.5 mm × 2 mm area in a 10 mm × 10 mm sample.

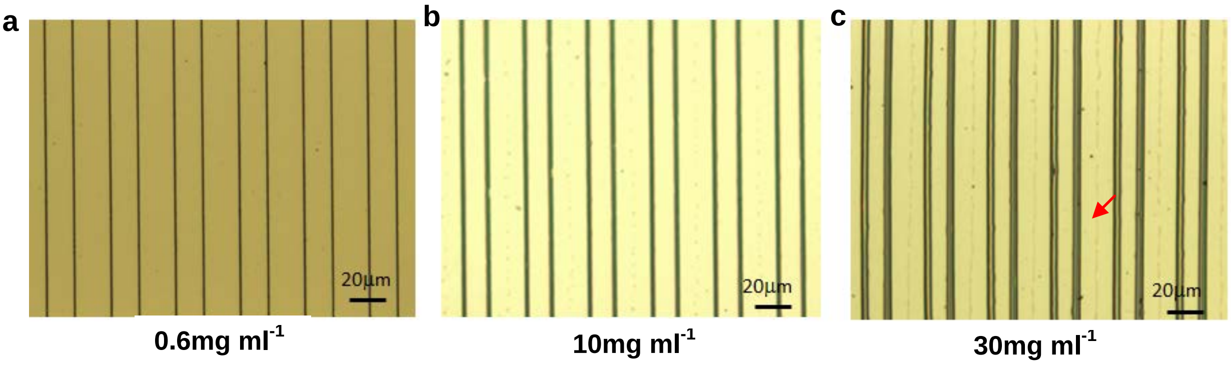

Supplementary Figure 3. Polystyrene patterns form solutions of different concentrations. (a, b, c) are for 0.6, 10 and 30 mg ml^{-1}, respectively. Patterns were generated from 1,2-dichlorobenzene solution of polystyrene with concentrations as indicated.

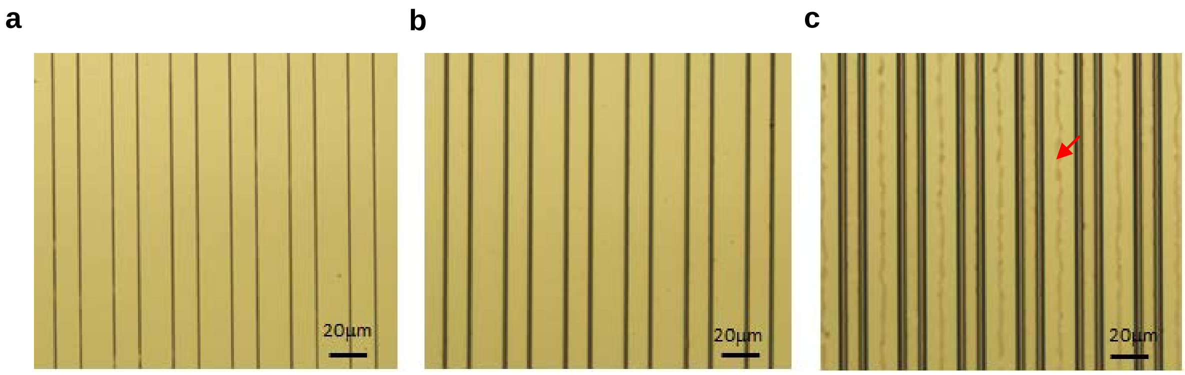

0.6mg ml^{-1} 10mg ml^{-1} 50mg ml^{-1}

Supplementary Figure 4. PVP patterns form solutions with different concentrations.

(a, b, c) are for 0.6, 10 and 50 mg ml^{-1}, respectively. Patterns were generated from IPA solution of PVP with concentrations as indicated.

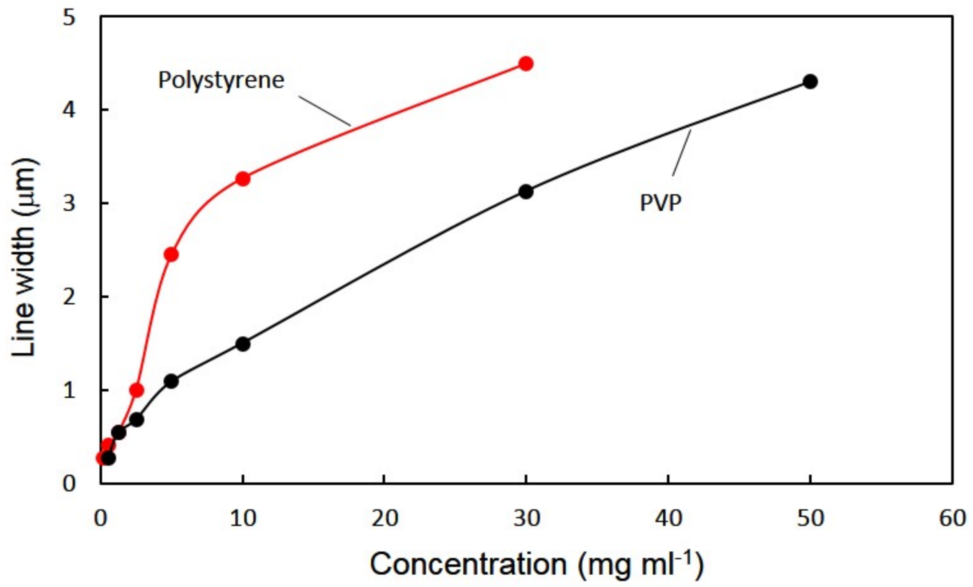

Supplementary Figure 5. Concentration dependence of line-width. The polystyrene lines were patterned from 1,2-dichlorobenzene solution and that for PVP from IPA solution.

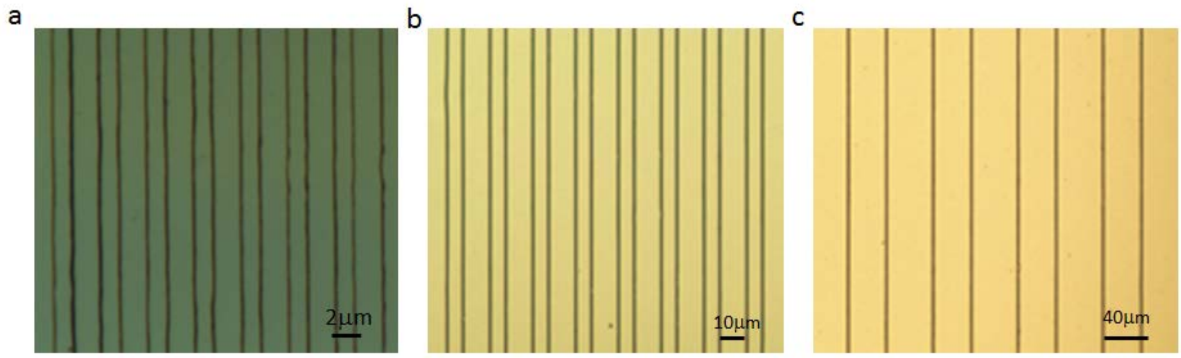

Supplementary Figure 6. Polystyrene structure variation with line separation. The polystyrene patterns were formed from 1, 2-dichlorobenzene solution (2.5 mg ml^{-1}).

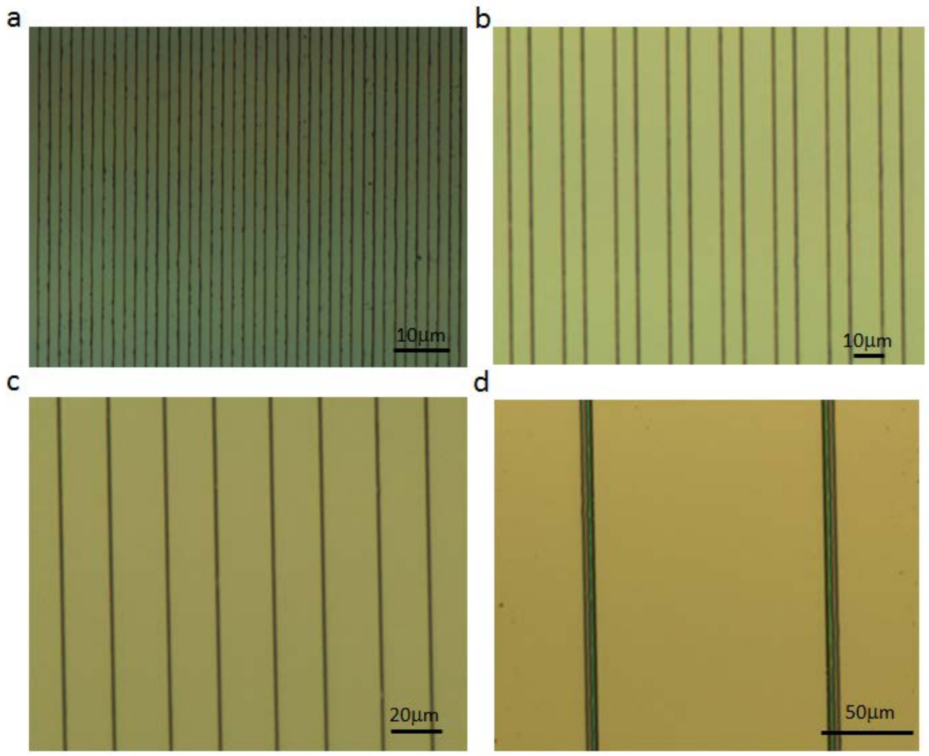

Supplementary Figure 7. PVP structure variation with line separation. The PVP patterns were formed from IPA solution (2.5 mg ml^{-1}).

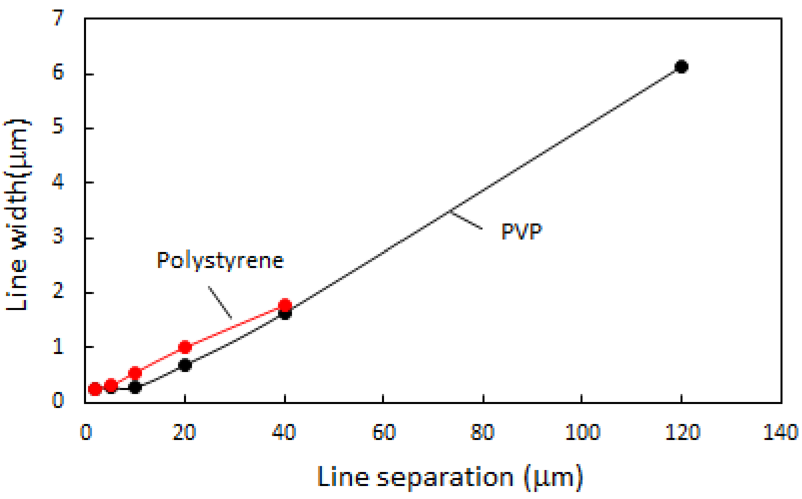

Supplementary Figure 8. Line-width variation with line separation. The polystyrene was patterned from 1,2-dichlorobenzene solution and that for PVP from IPA solution (2.5 mg ml^{-1}).

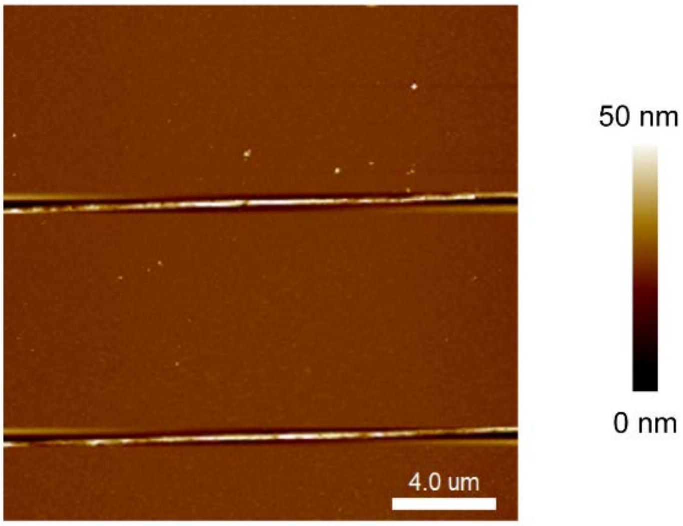

Supplementary Figure 9. AFM image of P(NDI2OD-T2) lines on SiO_2/Si substrate.

Process is detailed in Supplementary Note 1.

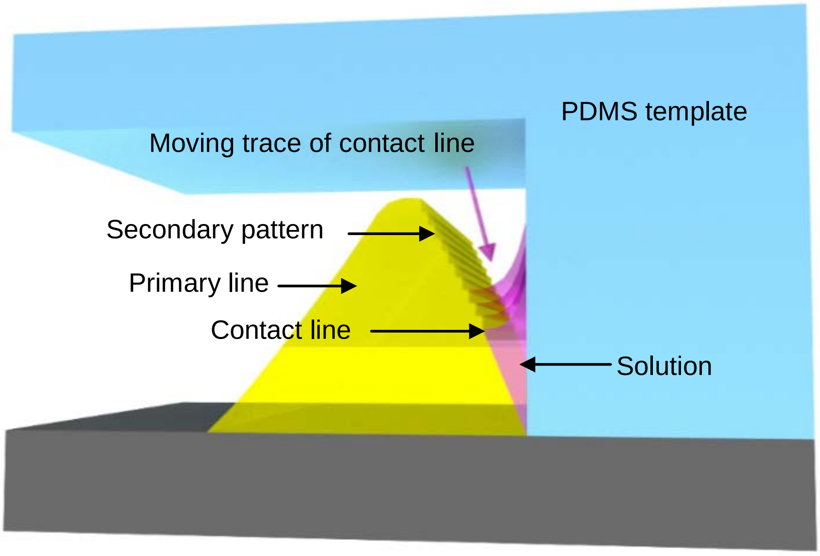

Supplementary Figure 10. Schematic illustration of nano-stripe formation mechanism. Process is detailed in Supplementary Note 2.

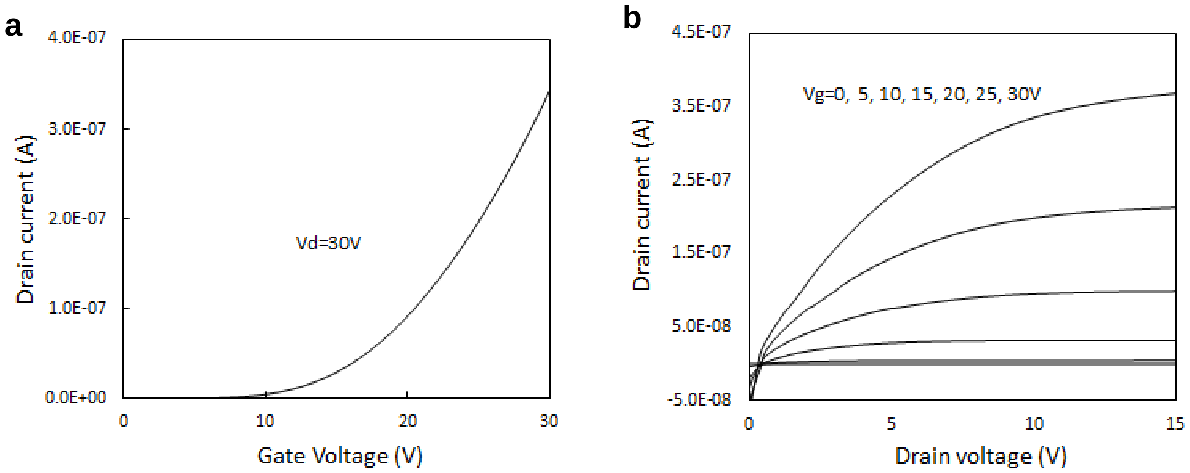

Supplementary Figure 11. Performance of spin-coated device. (**a**)Transfer and (**b**) output curves taken from a FET fabricated by spin-coated P(NDI2OD-T2). A 50-nm-thick P(NDI2OD-T2) film was spin-coated from toluene solution on a SiO_2(300 nm)/Si substrate with patterned Au electrodes. The subsequent fabrication process was identical to the transistor with patterned P(NDI2OD-T2) wires. The obtained charge mobility was 0.054 $cm^2\ V^{-1}s^{-1}$.

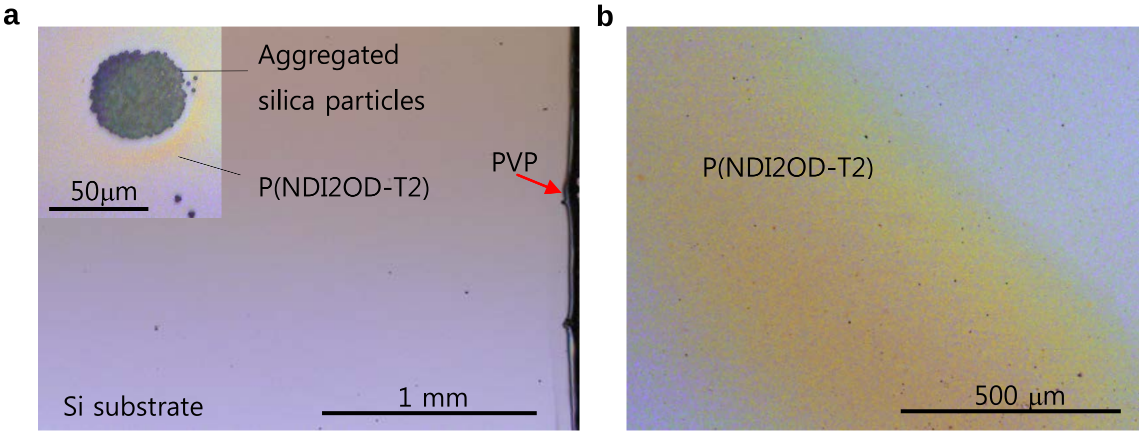

Supplementary Figure 12. Drying of polymers under un-patterned PDMS. (**a**) Image of dried PVP from IPA solution between PDMS and Si separated with 30μm polyimide film and image of dried P(NDI2OD-T2) from DCB between PDMS and Si substrate separated with silica beads with 1.5μm diameter [inset of (a)]. (**b**) Image of dried P(NDI2OD-T2) from DCB between PDMS and Si substrate with no spacers. Process is detailed in Supplementary Note 3.

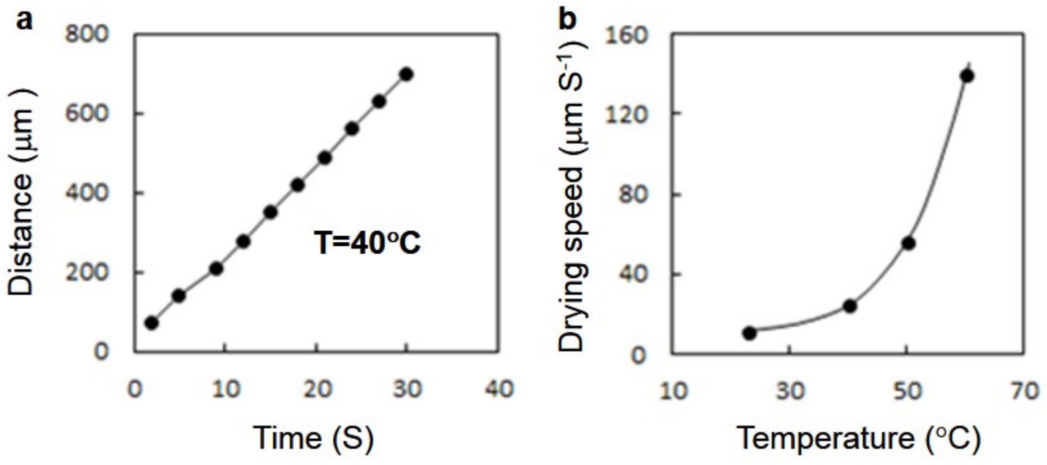

Supplementary Figure 13. Drying dynamics during line formation. (**a**) Time dependence of displacement of air-front measured at 40 °C and (**b**) the advancing speed of air-front at different temperatures. The curve shown in (b) was drawn only to guide the eyes. Process is described in Supplementary Note 4.

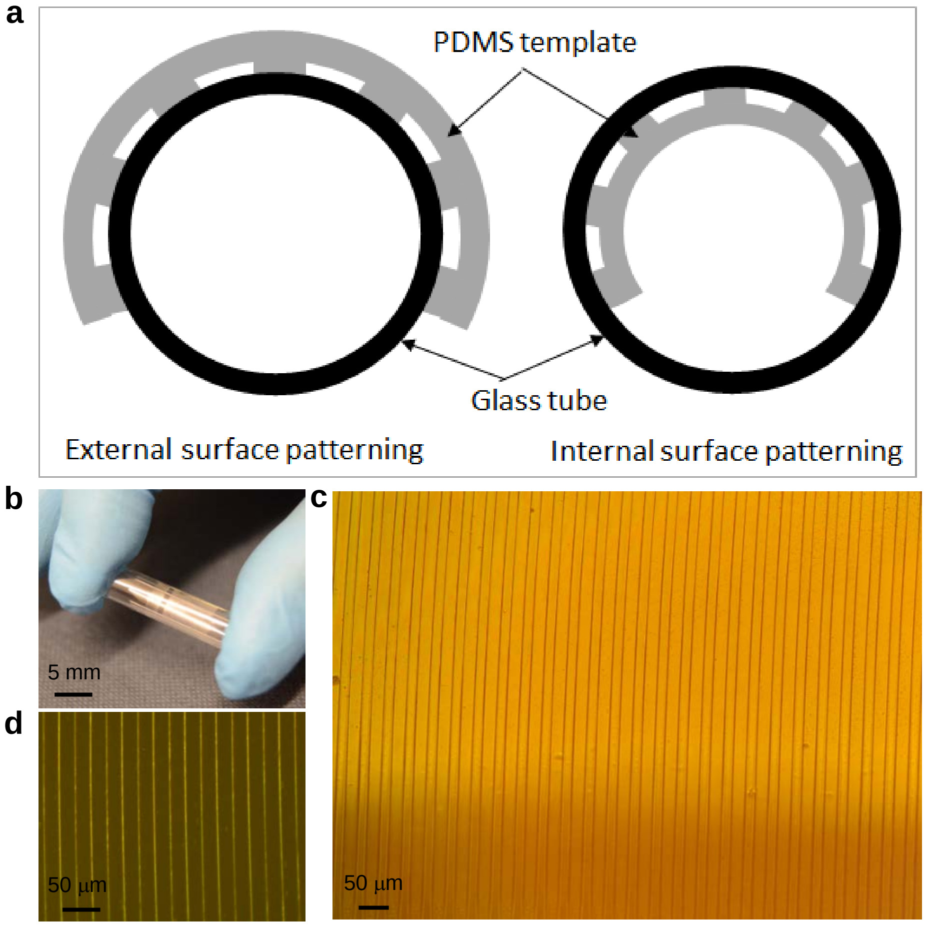

Supplementary Figure 14. Pattern generation on curved surfaces. (**a**) Schematic presentation of the process we used to fabricate polymer structure on both external (left) and inner (right) surfaces of tubes. (**b**)(**c**) Images of surface-patterned glass tube and polystyrene pattern on external surface. (**d**) Polystyrene lines patterned on inner surface of a glass tube. Process is detailed in Supplementary Note 5.

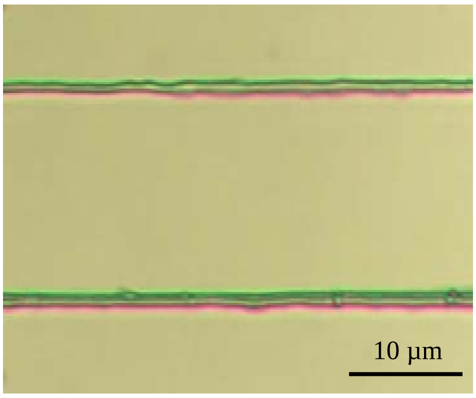

Supplementary Figure 15. DNA lines generated by our process. Process is detailed in Supplementary Note 6.

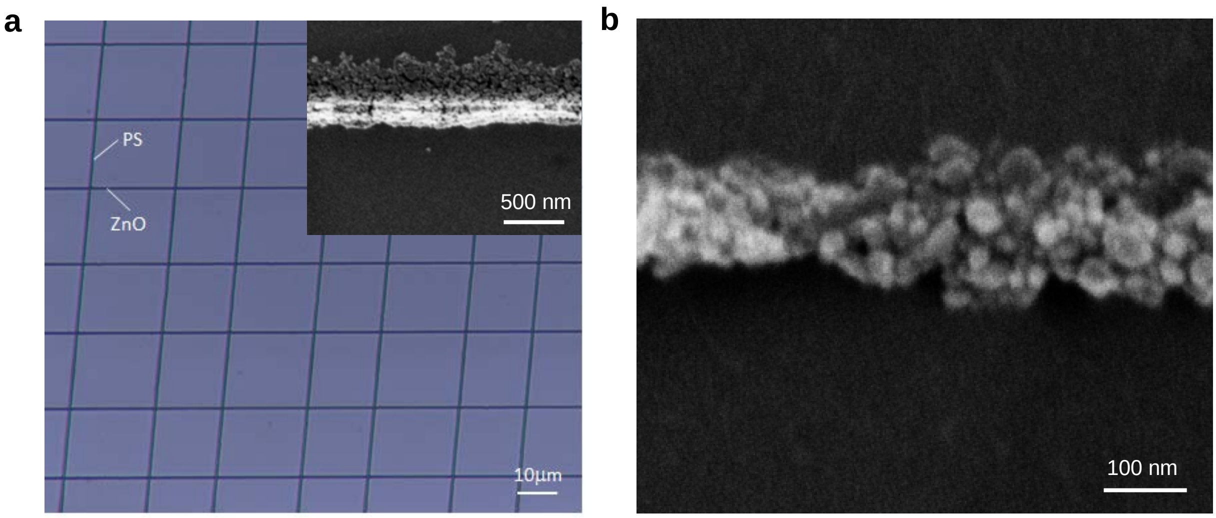

Supplementary Figure 16. Images of patterned ZnO nanoparticles. (**a**) ZnO particle lines (horizontal) patterned by using “spacer-applied” configuration with PS lines (vertical) as spacers (Inset is SEM image of a ZnO line). (**b**) SEM image of a ZnO line with 200 nm feature size. Process is detailed in Supplementary Note 6.

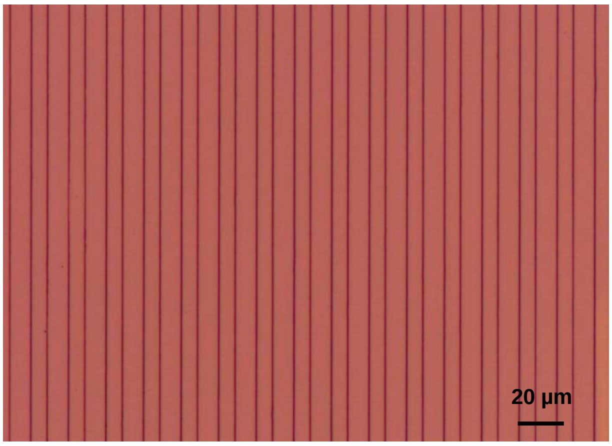

Supplementary Figure 17. PVP lines patterned with NES123L template. Process is detailed in Supplementary Note 7.

Supplementary Table 1. Comparison of the developed method with other techniques

Fabrication technique	Resolution	Aspect ratio	Patterning on curved surface	Thermal or irradiation	Direct patterning of particles	Reference
**Our work**	submicron meter	high	yes	no	yes	
**Conventional Photo-lithography**	micron meter	high	no	yes	no	[6,7]
**None-conventional Photolithography**	nano meter	high	no	yes	no	[8,9]
**E-beam lithography**	nano meter	high	no	yes	no	[7]
**Nanoimprint**	nano meter	high	no (except soft UV imprint)	yes	no	[10]
**Soft-lithography**	submicron meter	high	yes	no	yes	[11-13]
**Inkjet-printing**	micron meter	low	no	no	yes	[14,15]
**Screen-printing**	micron meter	high	yes	no	yes	[16,17]

Supplementary Note 1

EDX and AFM analysis

We have carried out energy dispersive x-ray (EDX) analysis to detect the residual polymer between the lines deposited on SiO_2/Si substrate and atomic force microscope (AFM) was used to check the dispersive residual. For the EDX analysis, while an obvious carbon peak was found with the patterned poly{[N,N0-bis(2-octyldodecyl)-naphthalene-1,4,5,8-bis(dicarboximide)-2,6-diyl]-alt-5,5'-(2,2'-bithiophene)} [P(NDI2OD-T2)] lines (C: 1.96 wt%, O: 26.87 wt%, Si: 71.17 wt%) only Si and O elements were detected between the lines (C: 0.00wt %, O: 27.08 wt%, Si: 72.92 wt%). Supplementary Figure 9 shows an AFM image of [P(NDI2OD-T2)] lines on the SiO_2 surface. The surface roughness measurements on both interline space of sample (0.360 nm) and unpatterned reference SiO_2/Si (0.303 nm) showed no obvious difference. The difference of the roughness taken from the sample and reference (0.57 Angstrom) is much less than the thickness of single layer of aromatic molecules (larger than 4 Angstroms).[1]

Supplementary Note 2

Formation of secondary nano-scale patterned lines

In addition to the line structure generated by groove pinning (referred to as primary lines), we also observed secondary, self-assembled stripe patterns with extremely high resolution (about 50 nm) on the walls of the primary lines. We attribute these structures to repeated pinning and de-pinning events during a late stage of drying. When the template ridges reached the substrate, a tiny amount of solution was trapped in the wedge-shaped space between a groove sidewall and the formed primary line next to it (Supplementary Figure 10).

Supplementary Note 3

Drying under unstructured PDMS template

We have conducted experiments with PDMS templates without grooves. Polymer solutions [P(NDI2OD-T2) in DCB (5mg ml^{-1}) or PVP in IPA(3mg ml^{-1})] were introduced into the space between a blank PDMS film and Si substrate which were separated by micron-meter-sized spacers. We found that with the solvent evaporation the solution aggregated towards the sample edges and spacers, and dried off there. No regular patterns were created on the substrate surfaces (Supplementary Figure 12a). In addition, drying solution between the PDMS and Si substrate without spacers has also been tested, and no regular line patterns were found (Supplementary Figure 12b). This further proves the importance of the grooves on the PDMS to pattern and pin the solution.

Supplementary Note 4

***In situ* observation of pattern formation**

Cutting the PDMS template along the direction perpendicular to the grooves in its structured area allows the air trapped from the external environment to be visualized with reflected light under a microscope. To observe the process of air trapping, PS was dried from a DCB solution on a silicon substrate using both the spacer-free and spacer-applied configurations.

We investigated air trapping in situ at various temperatures by drying PS from DCB solution on a silicon substrate with the spacer-free configuration. We measured the advancing speed of the air front at various temperatures in a series of samples. The linear relationship between the displacement of the air front and time revealed that the air font advanced with constant speed in the grooves (Supplementary Figure 13a). The advancing speed of the air front (V) and drying temperature T (in Kelvin scale) exhibited an exponential relationship (Supplementary Figure 13b):

$$V = V_0 \exp(-Q/kT).$$

Supplementary Note 5

Patterning on uneven surfaces

The developed method has a number of advantages over conventional fabrication techniques. Supplementary Figure 14a shows a schematic representation of the process we used to fabricate polymer structures on both curved external and inner surfaces. We used a 1mm-thick PDMS template to fabricate structures on both the external and inner surfaces of glass tubes with diameters of 6 mm. To pattern the external surface, 2 μl of a solution of polystyrene in 1,2-dichlorobenzene was dispensed onto the template surface. The template was then wrapped around the tube, fixed with scotch tape, and dried at room temperature. To pattern the inner surface, 2 μl of a solution of polystyrene in 1,2-dichlorobenzene was dispensed onto the inner surface of the tube, and a rolled template with its structured surface facing outward was then inserted into the tube and dried. Supplementary Figures 14b and Supplementary Figures 14c show images of surface-patterned glass tube and the polystyrene pattern on the external glass surface. A polystyrene line pattern on the inner surface of a glass tube is shown in Figure 14d.

Supplementary Note 6

Patterning of DNA molecules and nanoparticles

A major advantage of our process is the ability to pattern fragile materials that are not easily patterned using conventional methods that involve UV irradiation and/or heating. To demonstrate this ability to pattern fragile materials, we patterned DNA, which has interesting applications in pharmaceutical and diagnostic applications along with in the emerging area of DNA-based nano-assemblies of electronic devices.[2-4] We fabricated single-strand DNA (M-gene 20bp) with the sequence 5'-TCGGCTTTGAGGGGGCCTGA-3. Oxygen plasma was used to treat the PDMS template to improve its wettability with the aqueous phosphate buffer solution that DNA was dispersed in. The solution (2 µl) was dispensed onto the surface of a PDMS template, and a Si substrate was attached onto the wet surface and subsequently dried for 1 h at room temperature. Supplementary Figure 15 shows the obtained DNA lines. The colours of the lines are caused by thickness variation induced interference. The red/green colour domination of the DNA lines is originated from a strong absorption of short wavelength light. The DNA we have patterned is single-strand where purine and pyrimidine bases strongly absorb short-wavelength light.[5]

Nanoparticle patterning has also been demonstrated by patterning ZnO particles (around 50 nm size) from its colloidal suspension. The colloidal suspension was prepared by diluting a commercial ZnO particle suspension (40 wt% in ethanol, Sigma) to 1 wt% with IPA. Supplementary Figure 16a shows the ZnO lines (horizontal) patterned using the spacer-applied configuration with pre-patterned PS lines (vertical) as spacers. Supplementary Figure 16b shows an image of a ZnO particle line with a size of 200 nm, demonstrating the potential of our method for the high-resolution patterning of nanoparticles. In nanoparticle patterning, it is essential to use the spacer-applied configuration to minimize the trapping of particles under the template ridges. The spacer-free configuration causes residual particles to accumulate between the lines, resulting in poor pattern definition.

Supplementary Note 7

NEA123L template

We chose PDMS as the template material because its convenience of implementation. Other materials can be developed to optimize the pattern quality. For example, we used UV-curable Norland electronic adhesive material (NEA123L, Norland Products, Inc.) as an alternative template material. As-received NEA123L (4 µl) was drop-casted onto a cleaned PET substrate. A PDMS template with a surface area of 1 cm^2 was then gently laminated on the top of the viscous NEA123L with a small pressure so that the adhesive material spread uniformly over the entire surface of the PDMS template. The NEA123L was then curing with UV light (power density = 100 µJ cm^{-2}) for 5 min. After removing the PDMS template, the cured NEA123L film with the structure of the PDMS template was peeled off from the PET substrate. The NEA123L film was then laminated on a 2-mm-thick PDMS block by van der Waals force after cutting off its rough edge with a scalpel. Supplementary Figure 17 shows PVP patterned from IPA solution (1.25 mg ml^{-1}) on a Si substrate obtained using the NEA123L template.

Supplementary References

1 Shearer, C. J., Slattery, A. D., Stapleton, A. J., Shapter, J. G., Gibson, C. T. Accurate thickness measurement of graphene. *Nanotechnology* **27**, 124704(2016).

2 Kricka, L. J. Microchips, microarrays, biochips and nanochips: personal laboratories for the 21st century. *Clinica Chimica Acta* **307**, 219-223 (2001).

3 Seeman, N. C. DNA in a material world. *Nature* **421**, 427-432 (2003).

4 Yin, H., Brown, T., Wilkinson, J., Eason, R. & Melvin, T. Submicron patterning of DNA oligonucleotides on silicon. *Nucleic Acids Res.* **32**, e118 (2004).

5 D'Abramo, M., Castellazzi, C. L., Orozco, M., Amadei, A. *J. Phys. Chem. B* **117**, 8697-8704 (2013).

6 Madou, M. J. *Fundamentals of microfabrication: the science of miniaturization*. (CRC press, 2002).

7 Pimpin, A. & Srituravanich, W. Review on micro-and nanolithography techniques and their applications. *Engineering Journal* DOI:10.4186/ej.2012.16.1.37.

8 Rothschild, M. *et al.* Recent trends in optical lithography. *Lincoln Laboratory Journal* **14**, 221-236 (2003).

9 Fritze, M. *et al.* Subwavelength optical lithography with phase-shift photomasks. *Lincoln Laboratory Journal* **14**, 237-250 (2003).

10 Chou, S. Y., Krauss, P. R., Renstrom, P. J. Imprint lithography with 25-nanometer resolution. *Science* **272**, 85 (1996).

11 Pompe, T. *et al.* Submicron contact printing on silicon using stamp pads. *Langmuir* **15**, 2398-2401 (1999).

12 Xia, Y., Whitesides, G. M. Soft Lithography. *Annu. Rev. Mater. Sci.* **28**, 153-184(1998).

13 Santhanam, V., Andres, R. P. Microcontact printing of uniform nanoparticle arrays. *Nano Letters* **4**, 41-44 (2004).

14 Li, S., Chen, W., Chu, D. &` Roy, S. Self-Aligned High-Resolution Printed Polymer Transistors. *Adv. Mater.* **23**, 4107-4110 (2011).

15 Sirringhaus, H. *et al.* High-resolution inkjet printing of all-polymer transistor circuits. *Science* **290**, 2123-2126 (2000).

16 Lee, M. W., Lee, M. Y. & Song, C. K. Printing technologies for the gate and source/drain electrodes of OTFTs. *Journal of Information Display* **10**, 131-136 (2009).

17 Hyun, W. J., Secor, E. B., Hersam, M. C., Frisbie, C. D. & Francis, L. F. High-resolution patterning of graphene by screen printing with a silicon stencil for highly flexible printed electronics. *Adv. Mater.* **27**, 109-115 (2015).
