# Supplementary material for: High-resolution patterning of solution-processable materials via externally engineered pinning of capillary bridges
Source: Nat Commun. 2018 Jan 26;9:393. doi: 10.1038/s41467-018-02835-7 (PMC5786051; doi:10.1038/s41467-018-02835-7)
Supplement: Supplementary file 2 — Description of Additional Supplementary Files [file 41467_2018_2835_MOESM2_ESM.pdf]

Description of Additional Supplementary Files

File Name: Supplementary Movie 1
Description: Liquid splitting in micro-grooves during drying.

File Name: Supplementary Movie 2
Description: Liquid splitting in micro-grooves during drying (zoomed in).
